# VHH-Based Bispecific Antibodies Targeting Cytokine Production

**DOI:** 10.3389/fimmu.2017.01073

**Published:** 2017-09-01

**Authors:** Maxim A. Nosenko, Kamar-Sulu N. Atretkhany, Vladislav V. Mokhonov, Grigory A. Efimov, Andrey A. Kruglov, Sergei V. Tillib, Marina S. Drutskaya, Sergei A. Nedospasov

**Affiliations:** ^1^Engelhardt Institute of Molecular Biology, Russian Academy of Sciences, Moscow, Russia; ^2^Lomonosov Moscow State University, Moscow, Russia; ^3^Lobachevsky State University of Nizhny Novgorod, Nizhny Novgorod, Russia; ^4^National Research Center for Hematology, Moscow, Russia; ^5^German Rheumatism Research Center, Leibniz Institute, Berlin, Germany; ^6^Institute of Gene Biology, Russian Academy of Sciences, Moscow, Russia

**Keywords:** TNF, IL-6, macrophages, single-chain antibodies, V_H_H

## Abstract

Proinflammatory cytokines, such as TNF, IL-6, and IL-1, play pathogenic roles in multiple diseases and are attractive targets for biologic drugs. Because proinflammatory cytokines possess non-redundant protective and immunoregulatory functions, their systemic neutralization carries the potential for unwanted side effects. Therefore, next-generation anti-cytokine therapies would seek to selectively neutralize pathogenic cytokine signaling, leaving normal function intact. Fortunately, the biology of proinflammatory cytokines provides several such opportunities. Here, we discuss various applications of bispecific antibodies targeting cytokines with specific focus on selective TNF neutralization targeted directly to the surface of specific populations of monocytes and macrophages. These bispecific antibodies combine an anti-TNF V_H_H with V_H_Hs or scFvs directed against abundant surface molecules on myeloid cells and serve to limit the bioavailability of TNF produced by these cells. Such reagents may become prototypes of a novel class of anti-cytokine biologics.

Many currently used therapeutic antibodies represent antagonists or inhibitors of signaling cascades that are known to be pathogenic in a particular disease state. Examples include anti-cytokine therapies and immune checkpoint inhibitors, both of which have resulted in major advances in the treatment of autoimmune diseases and cancer. One common problem with many of such inhibitors, when applied systemically, is incomplete discrimination of “pathogenic” signaling from “physiological” signaling, the latter being beneficial for the patient. Therefore, most current therapies have unwanted side effects resulting from collateral damage to beneficial or protective signaling cascades. This problem can be potentially addressed through additional specificity conferred by more sophisticated inhibitory antibodies that target their cognate antigens only in a particular organ or cell lineage.

Therapeutic bispecific antibodies have showed efficacy in both experimental animal models and in clinical trials (1), finding applications in cancer immunotherapy (2) as well as in treatment of autoimmune diseases (3) and hemophilia (4). Examples include: (i) bispecific T-cell engagers (5) that redirect the activity of CD3+ cytotoxic T lymphocytes against CD19+ leukemias and lymphomas (6) and EpCAM+ solid tumors (7); (ii) bispecific NK-cell engagers that redirect the activity of CD16+ natural killer cells against CEA+ solid tumors (8); (iii) bispecific molecules composed of a CD19-binding moiety and an anti-CD47 immune checkpoint inhibitor, allowing for selective CD47 blockade on malignant B cells (9); (iv) bispecific molecules composed of an a β-secretase (BACE-1)-inhibiting moiety and an anti-transferrin receptor “trojan” moiety to facilitate permeation of the blood–brain barrier (10); and (v) bispecific molecules composed of an anti-HIV gp41 glycoprotein moiety and an anti-CD89 moiety, designed to facilitate virus clearance by blood neutrophils (11). Several designs of bispecific antibodies have been employed, including chemically conjugated monoclonal antibodies, quadroma-produced antibodies, or genetically fused recombinant single-chain Fvs (12). The lattermost molecules lack the Fc region and thus have very short serum half-lives. Recently, an interesting solution to the problem of rapid clearance of these molecules was proposed in the form of RNA delivery (13). Overall, more than a dozen bispecific antibodies have now been evaluated in clinical trials.

Several bispecific antibodies targeting cytokines have been described (14), allowing for dual cytokine blockade (15–18) as well as targeted cytokine neutralization on cytokine-producing cells (19) or at particular anatomical sites, such as inflamed joints (20). One important target in anti-cytokine therapy of autoimmune diseases is TNF, and many systemic anti-TNF biologics are approved for clinical use. There are several experimental reagents that have added a second specificity to an existing anti-TNF moiety. For example, a bispecific antibody directed against TNF and IL-17A was shown to be effective for the treatment of psoriasis (21). A TNF inhibitor with additional specificity to ROS-modified collagen allowed for targeted TNF inhibition in arthritic joints (20). Coppieters et al. (22) reported a highly efficient bispecific antibody that was able to bind TNF as well as an abundant serum protein (albumin), thus resulting in a significant increase of the antibody’s half-life *in vivo*. Two different inhibitors of TNFRI signaling, each with a second specificity to serum albumin for half-life extension *in vivo*, are effective in mouse models of Crohn’s disease and arthritis (23–25). Other studies have achieved longer half-lives and increased potencies of anti-TNF inhibitors by various types of dimerization or oligomerization (26–28) allowing the demonstration of their biological activity in mouse disease models. Although this was not directly determined, it may be assumed that all of these TNF inhibitors, including bispecifics, neutralized TNF produced by multiple cellular sources in a systemic fashion.

In our studies employing conditional gene targeting, we found that TNF produced by myeloid cells is pathogenic in several experimental mouse disease models (29–32). Assuming that TNF from other immune and non-immune sources may possess beneficial functions (33–35), we wanted to design an approach to pharmacologically limit TNF production only by myeloid cells. To this end, we designed, produced, and evaluated bispecific antibodies that bind TNF with one arm and engage surface molecules abundantly expressed on myeloid cells through another arm. Two such potential surface markers—F4/80 (EMR1, the product of the *Adgre1* gene) and CD11b (Mac-1a, Integrin αM, the product of the *Itgam* gene; expressed by myeloid cells, NK, and some other cells) can be employed.

The discovery of heavy-chain-only antibodies in *Camelidae* (36) led to the development of new technologies based on the ability to generate modular, high affinity binders (V_H_Hs) specific to almost any protein. One particular aspect that drew our attention was the usefulness of V_H_Hs in creating bispecific reagents, as two or even three V_H_Hs can be easily combined in a single polypeptide chain by the methods of genetic engineering (19) and expressed in prokaryotic systems. In order to specifically target TNF produced by myeloid cells, we have initially utilized a single-chain antibody to murine F4/80, which is exclusively expressed on myeloid cells with abundant expression on the surface of all mature macrophages (including microglia), Langerhans cells, and to a lesser degree on blood monocytes (37). We subsequently generated a novel V_H_H by immunizing a Bactrian camel with recombinant murine F4/80 and genetically fused it to an anti-hTNF V_H_H (19). Because of the specificity of this reagent to human TNF, all subsequent *in vitro* and *in vivo* experiments were performed using human TNF knock-in mice (38). Collectively, for all these bispecific antibodies, a term myeloid cell-specific TNF inhibitor (MYSTI) has been coined. Figure 1 outlines the design, purification, and experimental protocols for evaluation of these anti-TNF bispecific V_H_H-based reagents.

**Figure 1:**
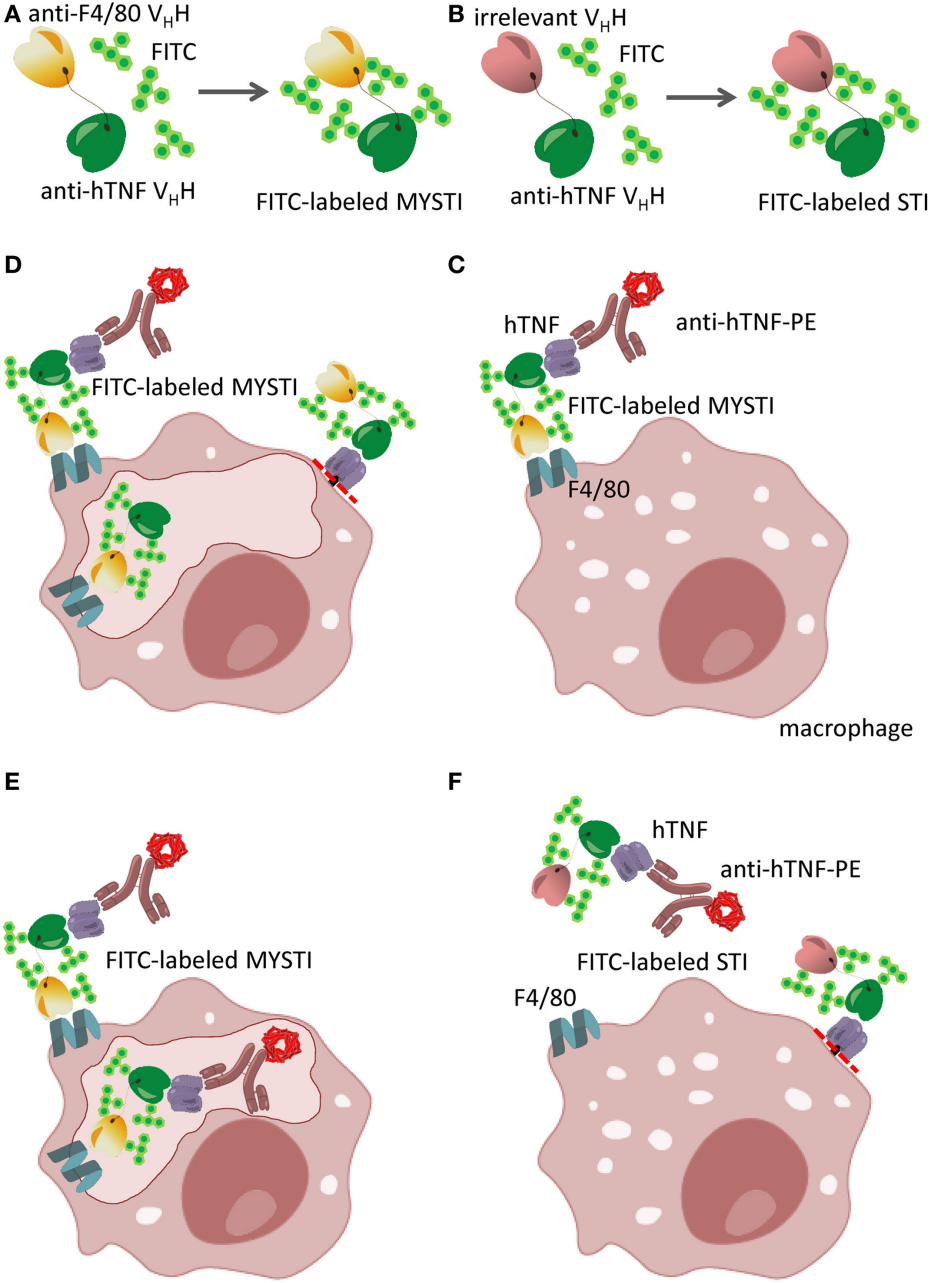
Schematic representation of bispecific anti-cytokine antibodies exemplified by myeloid-specific TNF inhibitors (MYSTI). **(A,B)** Generation of FITC-labeled bispecific antibody composed of anti-hTNF V_H_H and anti-F4/80 V_H_H (MYSTI, A) and control antibody composed of the same anti-hTNF V_H_H and irrelevant V_H_H [Systemic TNF Inhibitor, STI, **(B)**]. Briefly, antibodies were expressed and purified as previously described (19) and were subsequently labeled with FITC. Calculated F/P ratio was approximately four FITC molecules per protein molecule. **(C–F)** Schematic representation of MYSTI **(C–E)** and STI **(F)** binding to macrophages analyzed by flow cytometry and confocal microscopy. FITC-labeled MYSTI binds specifically to F4/80 on the surface of macrophages and can bind and retain exogenously added hTNF or hTNF produced by activated cells as detected by anti-hTNF phycoerythrin (PE)-labeled antibody (Miltenyi Biotec). This resulted in surface staining of macrophages both with FITC and PE **(C)**. MYSTI can be quickly internalized by macrophages resulting in intracellular FITC staining only **(D)**, or when hTNF was added exogenously—double staining for both FITC and PE **(E)**. STI did not bind to macrophages, as suggested by the absence of FITC or PE staining **(F)**. Red dotted line indicates the position of tmTNF cleavage by TACE (ADAM17). Adapted from (19).

Using flow cytometry, we found that MYSTI (exemplified here by MYSTI-2) binds to the surface of murine macrophages, competes with another anti-F4/80 reagent for this binding (Figures 2A,B), and attracts exogenously added human TNF to the surface of macrophages (Figure 2C). We then performed experiments to prove that endogenously produced TNF can also be retained on the cell surface. To this end, bone marrow-derived macrophages from humanized TNF knock-in (hTNF KI) mice (38) were incubated with MYSTI-2, or with control TNF-neutralizing antibodies lacking anti-F4/80 targeting module (referred here as systemic TNF inhibitor or STI), then washed and activated with LPS. As shown in Figure 2D, the amount of biologically active hTNF released into culture medium is significantly lower in the presence of MYSTI as compared to STI, suggesting that MYSTI indeed retained hTNF on the surface of macrophages and may limit its systemic release *in vivo*.

**Figure 2:**
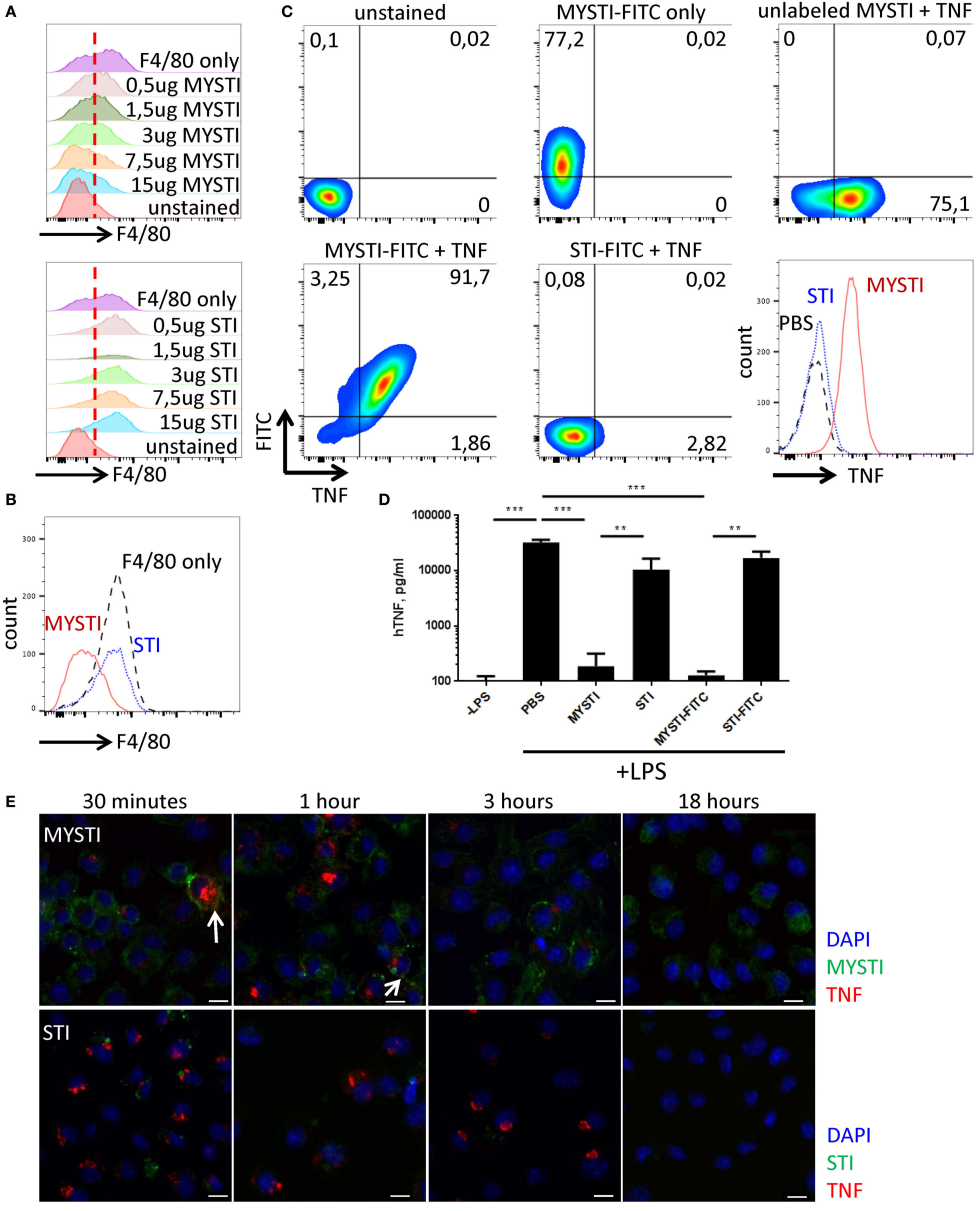

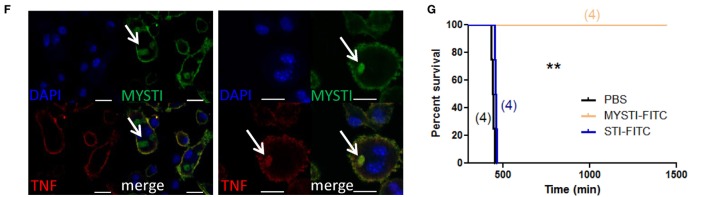
Characterization of MYSTI and STI interaction with macrophages *in vitro* and *in vivo*. **(A)** MYSTI, but not STI, competes with anti-F4/80 antibody for binding sites resulting in reduced staining for F4/80. Staining of macrophages with anti-F4/80 antibody in the presence of indicated concentrations of MYSTI (top panel) or STI (bottom panel). Red dotted line splits F4/80^−^ and F4/80^+^ cells on the left and on the right, respectively. Briefly, bone marrow-derived macrophages were simultaneously incubated with anti-F4/80 antibody (clone BM8 that competed for binding to F4/80 with anti F4/80 V_H_H, used in MYSTI) and indicated amounts of MYSTI or STI. All cells were gated as ViabilityDye^−^CD11b^+^. **(B)** Staining of macrophages with anti-F4/80 only or in the presence of MYSTI or STI. Data indicate that MYSTI selectively binds to F4/80. **(C)** MYSTI, but not STI, binds to the surface of macrophages and retains exogenously added hTNF. Surface staining of macrophages with MYSTI or STI and hTNF. Top row represents unstained or single stained cells as controls. Bottom row represents staining of macrophages with MYSTI-FITC and hTNF (left), STI-FITC and hTNF (middle), and a summarizing histogram of hTNF staining (right). Briefly, bone marrow-derived macrophages were subsequently incubated with MYSTI or STI followed by recombinant human TNF and with anti-hTNF antibody incubations. All cells were gated as VD^−^CD11b^+^. **(D)** MYSTI, but not STI, prevents hTNF release into the culture medium by LPS-stimulated macrophages. BMDM from hTNFKI mice were cultured with MYSTI or STI antibodies or PBS, washed once, and stimulated with 100 ng/ml of LPS from *E. coli*. Release of hTNF into culture medium was measured 4 h following induction with LPS using Ready-Set-Go ELISA kit (eBioscience). **p < 0.01; ***p < 0.001 in one-way ANOVA. **(E)** Dynamics of MYSTI and STI staining on LPS-activated macrophages as revealed by confocal microscopy. Briefly, macrophages were activated with 100 ng/ml of LPS for 3 h, followed by incubation with FITC-labeled MYSTI or STI for 15 min, then washed, and fixed at indicated time points. Fixed cells were consequently permeabilized and stained with anti-hTNF Ab labeled with PE. Starting from 30 min of incubation, MYSTI could be detected both on macrophage surface and inside the cells, while weak binding of STI was observed only after 30 min of incubation. Arrows show co-staining of MYSTI and anti-hTNF. Scale bars—10 μm. **(F)** MYSTI is internalized by macrophages. Confocal microscope images of macrophages stained with MYSTI (green), anti-hTNF (red), and counterstained with DAPI (blue). Briefly, cells were consequently incubated with MYSTI-FITC, recombinant hTNF, and anti-hTNF labeled with PE and then fixed. On each of the two images, top left part represents DAPI staining, top right—MYSTI-FITC, bottom left—anti-hTNF-PE, and bottom right—merged picture. Arrows show internalized MYSTI bound (right image) or not bound to hTNF (left image). Scale bars—20 μm. **(G)** FITC-labeled MYSTI retains its ability to protect mice in the model of LPS/D-Gal-induced hepatotoxicity. Briefly, mice were injected i.p. with 1.5 mg/kg, STI, or PBS and after 30 min were injected with lethal dose of LPS/D-Gal.

To get a better insight into the fate of hTNF and of MYSTI after its binding to the surface of macrophage, we utilized confocal microscopy, as outlined in Figures 1C–F. As expected, FITC-labeled MYSTI could stain these cells and was detected on the surface of activated macrophages as early as 15 min following incubation and—interestingly—up to 18 h later although in diminished amounts, consistent with our previous results (Figure 2E, top row and data not shown). In contrast, STI briefly stained activated macrophages after 15 min of incubation, while upon subsequent washing, such staining rapidly disappeared (Figure 2E, bottom row and data not shown). Since we did not detect binding of STI to unstimulated macrophages (data not shown), we hypothesized that such staining is due to recognition of transmembrane TNF (tmTNF) on the surface of activated macrophages. MYSTI was able to bind and retain human TNF produced by macrophages from hTNF KI mice (as indicated by the arrows in Figure 2E, top row) and exogenously added human TNF (Figure. 2F). We also detected rapid internalization of MYSTI (Figure 2F) starting from approximately 30 min of incubation with macrophages. Both unbound (Figure 2F, left) and TNF-bound (Figure 2F, right) bispecific antibodies were internalized, suggesting that internalization does not require TNF recognition by MYSTI. Exogenously added TNF, labeled by a secondary PE-conjugated antibody, could be detected on the surface of macrophages for at least 1–2 h (Figure 2F and data not shown).

Based on the encouraging finding that MYSTI, an antibody with two V_H_H domains, may be sufficiently long-lived on the surface of cytokine-producing cells, we evaluated these reagents *in vivo*. In LPS/D-Gal lethal toxicity model, pathogenic TNF is known to be produced by myeloid cells (39) and animals become moribund within 6–8 h (19). In this model, administration of MYSTI at 3 mg/kg completely protected mice, while the same dose of the control reagents (such as STI that contained exactly the same TNF-binding and neutralizing V_H_H module) failed to do so (19). Moreover, the results suggest that MYSTI retained its protective ability even at 1–1.5 mg/kg dose and modification with FITC did not affect its properties (Figure 2G and data not shown), thus allowing us to further investigate its fate *in vivo*. As an additional control, we used Infliximab as a systemic TNF inhibitor control, which also protected mice against LPS/D-Gal-induced hepatotoxicity at the dose of 1.5 mg/kg (data not shown); however, differences in molecular weight and avidity should be taken in account when comparing full-length systemic TNF-inhibitors with MYSTI. Additionally, MYSTI was active in anti-collagen antibody transfer arthritis model (data not shown). Another potential target for the “second specificity” is CD11b for which a V_H_H was recently reported (40). However, expression of this molecule is not strictly restricted to myeloid cells (41, 42) and, additionally, F4/80 appears to be expressed at significantly higher levels, as compared to CD11b [according to mass spectrometric database (43)].

## Concluding Remarks and Future Perspectives

The remarkable success of anti-cytokine therapy in treating autoimmune and other diseases suggests that bispecific antibodies targeting pro-inflammatory cytokines, such as TNF or IL-6, will be developed and used. V_H_H technology has provided attractive antigen-binding modules for such bifunctional antibodies that simplify their engineering, expression, and purification. The central issue here is the nature of the “second specificity.” These may include additional anti-cytokine moieties or binding modules directing these reagents to either specific organs or cell types. Our own studies explored the possibility of targeting anti-cytokine antibodies to the surface of specific TNF-producing cell types, as we believe that some cells represent predominantly pathogenic sources of cytokine, at least in a particular disease or disease state. We continue to evaluate the features of selective TNF inhibitors with a focus on their *in vivo* ability to bind and neutralize TNF produced by myeloid cells, but not by other cell types. We aim to expand this concept to other pro-inflammatory cytokines, such as IL-6, using V_H_Hs generated against human IL-6 (44), although the safety of myeloid-specific IL-6 inhibitors needs to be assessed with regards to IL-6’s role in the development of lymphocytes (45). This approach is a pharmacological analog of inducible cell type-restricted gene ablation *in vivo*, with the advantage that the effects of antibodies are reversible and more relevant for preclinical evaluation. Although ongoing studies are mostly performed in animal models, one may expect that some of these V_H_H-based multispecific biologics will be eventually approved for human therapy, as has already happened for several such reagents utilizing more conventional antigen-binding modules, such as scFv.

## Ethics Statement

All manipulations with animals were carried out in accordance with recommendations in the Guide for the Care and Use of Laboratory Animals (NRC 2011), the European Convention for the Protection of Vertebrate Animals Used for Experimental and Other Scientific Purposes, Council of Europe (ETS 123), and “The Guidelines for Manipulations with Experimental Animals” (the decree of the Presidium of the Russian Academy of Sciences of April 02, 1980, no. 12000-496). All animal procedures were approved by Scientific Council of the Engelhardt Institute of Molecular Biology.

## Author Contributions

MN, K-SA, GE, AK, MD, and SN designed the research and analyzed the data; MN, K-SA, and MD performed the experiments; VM, GE, and ST developed and produced bispecific reagents; all authors contributed to writing the manuscript.

## Conflict of Interest Statement

GE, AK, and SN are coauthors of the patent application describing the initial version of MYSTI. Other authors declare no conflict of interest.
